# Alcohol use and family-related factors among Spanish university students: the unHicos project

**DOI:** 10.1186/s12889-022-13900-8

**Published:** 2022-08-19

**Authors:** Esperanza Romero-Rodríguez, Carmen Amezcua-Prieto, María Morales-Suárez-Varela, Carlos Ayán Pérez, Ramona Mateos-Campos, Alba Marcos-Delgado, Rocío Ortíz-Moncada, Susana Redondo Martín, Carmen Rodríguez-Reinado, Miguel Delgado-Rodríguez, Gemma Blázquez Abellán, Jessica Alonso Molero, Sandra Martín-Peláez, José M. Cancela-Carral, Luis F. Valero Juan, Virginia Martínez-Ruiz, Tania Fernández-Villa

**Affiliations:** 1 Maimonides Biomedical Research Institute of Cordoba / Reina Sofia University Hospital / University of Córdoba, Córdoba, Spain; 2 Consortium for Biomedical Research in Epidemiology and Public Health (CIBER Epidemiología Y Salud Pública-CIBERESP), Madrid, Spain; 3 Department of Preventive Medicine and Public Health, University of Granada, Granada, Spain; 4 Instituto de Investigación Biosanitaria (Ibs), Granada, Spain; 5 Department of Preventive Medicine and Public Health, Food Sciences, Toxicology and Legal, Medicine, Area of Preventive Medicine and Public Health, University of Valencia, Valencia, Spain; 6 Biomedical Research Center Network on Epidemiology and Public Health (CIBERESP), Institute of Health Carlos III, Avenida Monforte de Lemos, 3-5, Pabellón 11, Planta 0, 28029 Madrid, Spain; 7 Department of Special Didactics, Galicia Sur Health Research Institute (IIS Galicia Sur), Sergas-UVIGO, HealthyFit Research Group, University of Vigo, Vigo, Spain; 8 Departament of Biomedical and Diagnostic Sciences. Area of Preventive Medicine and Public Health, University of Salamanca, Salamanca, Spain; 9 Faculty of Health Sciences, Department of Biomedical Sciences, Area of Preventive Medicine and Public Health, Universidad de León, Ponferrada Campus S/N, 24401 Ponferrada, Léon, Spain; 10 Institute of Biomedicine (IBIOMED), The Research Group in Gene - Environment and Health Interactions, Universidad de León, León, Spain; 11 Department of Community Nursing, Preventive Medicine and Public Health and History of Science, Food and Nutrition Research Group, Area of Preventive Medicine and Public Health, University of Alicante, Alicante, Spain; 12 Department of Preventive Medicine and Public Health, University of Valladolid, Valladolid, Spain; 13 Centro de Investigación en Recursos Naturales, Salud Y Medio Ambiente, Universidad de Huelva, Huelva, Spain; 14 Department of Health Sciences, Area of Preventive Medicine and Public Health, University of Jaen, Jaen, Spain; 15 Department of Medical Sciences, Area of Preventive Medicine and Public Health. Faculty of Pharmacy, University of Castilla La Mancha, Albacete, Spain

**Keywords:** Alcohol, University Student, Binge Drinking, AUDIT

## Abstract

**Background:**

During adolescence and youth there are relevant changes in the consolidation, gain or loss of consumption habits and lifestyles and the family factors has a fundamental role to development these habits. The study of the consumption of toxins, such as alcohol intake, is crucial at this stage due to the repercussions that said consumption presents in adulthood. Therefore, the objective of our study was to evaluate the associations between alcohol consumption patterns and related family factors (family functioning, family history of alcohol consumption) in Spanish university students.

**Methods:**

Observational, descriptive, cross-sectional, multicenter study, carried out in first-year university students from 11 Spanish universities. Through an online questionnaire, alcohol consumption (risky consumption and intensive consumption or binge drinking), family functioning and history of alcohol in the family were evaluated.

Risky alcohol consumption and binge drinking were assessed using the AUDIT test, and family functioning was assessed using the family APGAR questionnaire. A descriptive analysis of the data was performed, as well as the Chi-Square test and Student's T-Test, and non-conditional logistic regression models were carried out to examine this association.

**Results:**

The prevalence of risky alcohol consumption identified in the 10,167 respondents was 16.9% (95% CI = 16.2–17.6), and that of BD was 48.8% (95% CI = 47.9–48.8). There is a significant association between risky alcohol consumption and family functioning in students of both sexes, with greater consumption in the face of severe dysfunctional support (men OR = 1.72; *p* < 0.001 and women OR = 1.74; *p* < 0.001) and family history of consumption (*p *= 0.005). Regarding the binge drinking pattern, no statistically significant differences were observed.

**Conclusions:**

Risky alcohol consumption in university students is associated with dysfunctional family support, unlike the binge drinking pattern, where there is no such association.

The findings of this study show the importance of creating prevention programs focused on the family approach in university students, which include alcohol screening in the population with a family history of this substance, and greater social support from health services.

**Supplementary Information:**

The online version contains supplementary material available at 10.1186/s12889-022-13900-8.

## Introduction

Alcohol consumption is one of the risk factors for death and disability [1]. The World Health Organization (WHO) estimates that 5.3% of deaths and 5.1% of morbidity worldwide are due to alcohol [2]. According to the latest Survey on Alcohol and Drugs in Spain (EDADES), 85.1% of the population between 15 and 24 years old reported having consumed alcohol at some point in their life and 59.7% stated that they had ingested alcohol in the last month, the consumption more prevalent in men (64.4%) than in women (54.8%) [3].

During adolescence and youth there are relevant changes in the reinforcement, gain or loss of consumption habits and lifestyles. The study of consuming toxins, such as alcohol intake, is crucial at this stage due to the repercussions that this consumption presents in adulthood [4–6].

The process of personal maturation, entering university or the employment world and the family or social environment are determining factors in strengthening these habits. Specifically, familial socialization makes up the basis of an individual’s personality, attitude, values and self-concept development [7].

A functional family is one that offers safety, cohesion, communication and routine expression of positive affection and is based on a shared set of cultural norms and values [8]. Good family functioning allows adaptation to the changes that occur during growth [9].

Families who use drugs substances such as alcohol tend to be characterized by low levels of cohesion, low tolerance for frustration, unrealistic expectations of children, role reversal, isolation, and poor parenting skills, characteristics associated with adverse consequences for the families [7].

Some international studies have linked family history of alcohol consumption and the history of a dysfunctional family with a greater probability of risky alcohol consumption in the young population [10–14].

However, despite the relevance of the family in the approach to alcohol consumption in the population and in an individual’s development, especially in university students, there is little evidence of the association that family factors have regarding the patterns of alcohol consumption in university students. Therefore, the objective of our study was to examine the associations between alcohol consumption patterns and associated family factors (family functioning and history of alcohol consumption) in first-year university students.

## Methods

### Design and study sample

This study is an observational, cross-sectional analysis of a dynamic cohort of university students enrolled in different degrees in the first year of study from eleven Spanish public universities (Alicante, Cantabria, Castilla la Mancha, Granada, Huelva, Jaén, León, Salamanca, Valladolid, Valencia and Vigo) that are part of the uniHcos (University, Life Habits, Follow-up Cohort) project [15], which aims to evaluate the habits and lifestyles of Spanish university students. The study received approval from the Ethics Committee of the University of León (Code: ETICA-ULE-007–2016).

Selection criteria: 1) Be a first-year university student enrolled in a Spanish university included in the uniHcos project; 2) Complete the self-administered form and grant informed consent for participation in the study.

Since the uniHcos project is a dynamic cohort, we did not determine a minimum sample size for this study.

### Data collection

Students who met the selection criteria received an email through their university account that included information about the uniHcos project and a link to a mandatory informed consent form that had to be completed before answering the study questionnaire. Students interested in participating completed a self-reported ad-hoc online questionnaire between October 2011 and March 2018 using the SphinxOnline® platform. The questionnaire included questions on alcohol consumption from the National Health Survey (ENS) [16] and the EDADES survey [3].

Two patterns of alcohol consumption were analyzed: risky consumption and intensive consumption or binge drinking (BD). Both patterns were assessed using the Alcohol Use Disorders Identification Test (AUDIT) [17]. This questionnaire has been validated in this population by Kokotailo et al. [18] and Verhoog et al. [19]. Heavy alcohol consumption, or BD, was defined as the intake of 6 or more alcoholic beverages in a single session, for both men and women. Patients with an AUDIT score ≥ 8 were considered to have risky alcohol consumption. This score determines the risk of developing alcohol consumption problems.

Family functioning was assessed using the family APGAR questionnaire [20], a 5-item questionnaire that measures five domains: "Adaptation", "Partnership", "Growth", "Affection" and "Resolve". Each item is scored on a 3-point scale: almost always (0 points), sometimes (1 point), and almost never (2 points). The sum can be from zero to ten points and families can be characterized as: a functional family (7–10) or a dysfunctional family (≤ 6). The dysfunctional family can be classified as mild (> 2 and < 7) or severely dysfunctional (≤ 2).

The questionnaire was validated in Spanish by the Bellon et al. group [21].

### Data analysis

A descriptive analysis was performed where measures of central tendency (mean and median) and dispersion (standard deviation and range) of the quantitative variables and prevalence of the qualitative variables were calculated.

To evaluate the relationship between the factors associated with alcohol consumption and the dependent variables (risky consumption and BD), we used the Chi-Square test and T-Student Test, as well as unconditional logistic regression analysis. Odds Ratio (OR) values and their respective 95% confidence interval (CI) were found for each variable. All models were stratified by sex and adjusted for age, occupation, type of residence, family support, and family history of alcohol consumption. Statistical analysis was performed using the IBM Statistic SPSS 20 program with a significance level of 95% (*p* = 0.05).

## Results

A total of 10,167 participants completed the questionnaire, 72.2% of which were women (95% CI: 70.9–77.2). The mean age of the subjects was 22.1 years (SD: 4.5; limits: 17–63; 95% CI 20.0–20.2). Students made up 66.2% (95% CI: 65.3–67.2) of the respondents, 10.7% (95% CI: 10.1–11.3) combined study and work, and 23.1% (95% CI: 22.3–24.0) were students and looking for work. Of those surveyed, 48% live in the family home or in their own home and 39% in a rented apartment. Table 1 shows the main sociodemographic and occupational characteristics of the participants by sex, with significant differences observed in relation to age (*p* < 0.001), occupation (*p* < 0.001), place of residence (*p* < 0.001), university degree (*p* < 0.001), first-choice degree (*p* < 0.001), and university location (*p* < 0.001). The overall response rate obtained was 4.0%.

**Table 1:** Sociodemographic and occupational characteristics of the sample (*n* = 10,167)

Socioeconomic and occupational characteristics		Men (*n* = 2823) n (%)	Women (*n* = 7344) n (%)	*p*
**Age (years)**	17–20	2073 (73.4)	5737 (78.1)	< 0.001
	21–24	443 (15.7)	1053 (14.3)	
	≥ 25	307 (10.9)	554 (7.5)	
**University**	Alicante	245 (8.7)	609 (8.3)	< 0.001
	Cantabria	29 (1.0)	59 (0.8)	
	Castilla La Mancha	61 (2.2)	131 (1.8)	
	Granada	806 (28.6)	2130 (29.0)	
	Huelva	107 (3.8)	321 (4.4)	
	Jaén	75 (2.7)	215 (2.9)	
	León	224 (7.9)	676 (9.2)	
	Salamanca	353 (12.5)	858 (11.7)	
	Valencia	346 (12.3)	1106 (15.1)	
	Valladolid	204 (7.2)	412 (5.6)	
	Vigo	373 (13.2)	827 (11.3)	
**Field**	Art and Humanities	261 (9.2)	964 (13.1)	< 0.001
	Science	515 (18.2)	1041 (14.2)	
	Health Sciences	506 (17.9)	1762 (24.0)	
	Social and Legal Sciences	900 (31.9)	3161 (43.0)	
	Engineering and Architecture	641 (22.7)	416 (5.7)	
**University degree**	Yes	565 (20.0)	1717 (23.4)	< 0.001
	No	2258 (80.0)	5627 (76.6)	
**Residence**	University housing	326 (11.5)	971 (13.2)	< 0.001
	Family /own home	1470 (52.1)	3407 (46.4)	
	Rented apartment	1027 (36.4)	2966 (40.4)	
**Occupation**	Student	580 (20.5)	1772 (24.1)	< 0.001
	Student and employed	344 (12.2)	741 (10.1)	
	Student looking for work	1899 (67.3)	4831 (65.8)	
**Characteristics and patterns of alcohol consumption**		Men n (%)	Women n (%)	*p*
** Age at start of consumption (years)**	< 13	348 (13.2)	922 (13.4)	0.003
	14–15	1040 (39.4)	2978 (43.2)	
	16–17	1020 (38.6)	2411 (35.0)	
	≥ 18	234 (8.9)	587 (8.5)	
** Drinking place**	Public place			0.113
	Bar/Restaurant	948 (33.6)	2395 (32.6)	
	University celebration	1246 (44.1)	3249 (44.2)	
	Public street	88 (3.1)	307 (4.2)	
	Private place			
	Private celebration	174 (6.2)	480 (6.5)	
	Other	367 (13.0)	913 (12.4)	
** Risky alcohol consumption**	No	2188 (77.5)	6262 (85.3)	< 0.001
	Yes	635 (22.5)	1082 (14.7)	
** Binge drinking**	No	1484 (52.6)	3719 (50.6)	0.082
	Yes	1339 (47.4)	3625 (49.4)	
** Family history of alcohol use**	**0**	**2088 (74.0)**	**5333 (72.6)**	** < 0.001**
	1 or more	735 (26.0)	2011 (27.4)	

The prevalence of risky alcohol consumption in the surveyed population was 16.9% (95% CI = 16.2–17.6), 22.5% (95% CI = 21.0–24.0) in men and 14.7% (95% CI = 13.9–15.5) in women. The prevalence of BD was 48.8% (95% CI = 47.9–48.8), 47.4% (95% CI = 45.6–49.3) in men and 49.4% (95% CI = 48.2–50.5) in women.

Table 2 shows the prevalence of the respondents’ family history in terms of the socio-demographic and occupational variables, stratified by sex. Significant differences are observed in risky alcohol consumption and the family history of alcohol in the father (0.034), the mother (< 0.001), the child (0.002), the partner (0.017), the uncle (0.013) and the number of relatives overall (*p* = 0.005). In terms of BD consumption, significant differences were only obtained if there was a history of consumption in the couple (0.025).

**Table 2:** Alcohol consumption patterns and family history of alcohol in Spanish university students

		**Students with risky alcohol consumption**			**Students with Binge drinking**		
**Family members with alcohol consumption**		No (*n* = 8450) n (%)	Yes (*n* = 1717) n (%)	*P*	No (*n* = 5203) n (%)	Yes (*n* = 4964) n (%)	*P*
**Father**	No	7832 (92.7)	1566 (91.2)	0.034	4800 (92.3)	4598 (92.6)	0.478
	Yes	618 (7.3)	151 (8.8)		403 (7.7)	366 (7.4)	
**Mother**	No	8346 (98.8)	1686 (98.2)	< 0.001	5140 (98.8)	4892 (98.5)	0.291
	Yes	104 (1.2)	31 (1.8)		63 (1.2)	72 (1.5)	
**Son/Daughter**	No	8449 (100)	1714 (99.8)	0.002	5200 (99.9)	4963 (100.0)	0.340
	Yes	1 (0.0)	3 (0.2)		3 (0.1)	1 (0.0)	
**Grandfather/** **Grandmother**	No	7873 (93.2)	1577 (91.8)	0.051	4836 (92.9)	4614 (92.9)	0.996
	Yes	577 (6.8)	140 (8.2)		367 (7.1)	350 (7.1)	
**Sibling**	No	8352 (98.8)	1690 (98.4)	0.157	5142 (98.8)	4900 (98.7)	0.593
	Yes	99 (1.2)	27 (1.6)		61 (1.2)	64 (1.3)	
**Partner**	No	8441 (99.9)	1711 (99.7)	0.017	5197 (99.8)	4961 (99.9)	0.025
	Yes	9 (0.1)	6 (0.3)		12 (0.2)	3 (0.1)	
**Uncle/Aunt**	No	7459 (88.3)	1481 (86.3)	0.019	4584 (88.1)	4356 (87.8)	0.587
	Yes	991 (11.7)	236 (13.7)		619 (11.9)	608 (12.2)	
**Number of family members**	0	6215 (73.6)	1206 (70.2)	0.005	3789 (72.8)	3632 (73.2)	0.697
	1 or more family members	2235 (26.4)	511 (29.8)		1414 (27.2)	1332 (26.8)	

Table 3 shows the relationship between risky alcohol consumption and the family support received by the participants. Significant differences were obtained regarding adaptation (*p* < 0.001; greater consumption with less adaptation), partnership (*p* < 0.001; greater consumption with less partnership), growth (*p* < 0.001, greater consumption with less growth), affection (*p* < 0.001; higher consumption with less affection), resolve (*p* < 0.001; higher consumption with lower resolution), and the family APGAR questionnaire score (*p* < 0.001; higher consumption in the presence of severe dysfunctional family support).

**Table 3:** Patterns of alcohol consumption and family functioning among Spanish university students

Family functioning (Family APGAR questionnaire)		Risky alcohol consumption			Binge drinking		
		No (*n* = 8450) n (%)	Yes (*n* = 1717) n (%)	P	No (*n* = 5203) n (%)	Yes (*n* = 4964) n (%)	*P*
**Adaptation**	Almost never	482 (5.7)	421 (24.5)	< 0.001	1129 (21.7)	1060 21.4)	0.230
	Sometimes	1768 (20.9)	139 (8.1)		337 (6.5)	284 (5.7)	
	Almost always	6200 (73.4)	1157 (67.4)		3737 (71.8)	3620 (72.9)	
**Partnership**	Almost never	1269 (15.0)	337 (19.6)	< 0.001	1595 (30.7)	1704 (34.3)	< 0.001
	Sometimes	2678 (31.7)	621 (36.2)		835 (16.0)	771 (15.5)	
	Almost always	4503 (53.3)	759 (44.2)		2773 (53.3)	2489 (50.1)	
**Growth**	Almost never	1391 (16.5)	650 (37.9)	< 0.001	1768 (34.0)	1870 (37.7)	< 0.001
	Sometimes	2988 (35.4)	404 (23.5)		893 (17.2)	902 (18.2)	
	Almost always	4071 (48.2)	663 (38.6)		2542 (48.9)	2192 (44.2)	
**Affection**	Almost never	1226 (14.5)	736 (42.9)	< 0.001	2052 (39.4)	2061 (41.5)	< 0.001
	Sometimes	3377 (40.0)	326 (19.0)		770 (14.8)	782 (15.8)	
	Almost always	3847 (45.5)	655 (38.1)		2381 (45.8)	2121 (42.7)	
**Resolve**	Almost never	147 (1.7)	239 (13.9)	< 0.001	620 (11.9)	542 (10.9)	0.014
	Sometimes	923 (10.9)	35 (2.0)		109 (2.1)	73 (1.5)	
	Almost always	7380 (87.3)	1143 (84.0)		4474 (86.0)	4349 (87.6)	
**Total score on the Family APGA** **Questionnaire**	Normal functioning	6160 (72.9)	1120 (65.20)	< 0.001	3721 (71.5)	3559 (71.7)	0.308
	Mild dysfunction	1615 (19.1)	395 (23.0)		1013 (19.5)	997 (20.1)	
	Severe dysfunction	675 (8.0)	202 (11.8)		469 (9.0)	408 (8.2)	

Table 4 shows the results of the unadjusted and the adjusted logistic regression analyses between risky alcohol consumption and sociodemographic, occupational and family characteristics, stratified by sex. The adjusted analysis shows that risky alcohol consumption in men was significantly related to age (aOR = 1.67; *p* = 0.007; with higher consumption in respondents aged 21–24 years), place of residence (aOR = 0.58; *p* < 0.001 lower consumption in those in a rented apartment), occupation (aOR = 1.64; *p* = 0.001; higher consumption in those studying and working), and family functioning (aOR = 1.72; *p* < 0.001; higher consumption in those with severe family dysfunction). In addition, the adjusted analysis reveals that risky alcohol consumption in women was related to age (aOR = 1.72; *p* = 0.001; with higher consumption in respondents aged 17–21 years), place of residence (aOR = 0.54; *p* < 0.001; lower consumption in those in a rented apartment), occupation (aOR = 1.64; *p* = 0.001; higher consumption in those studying and looking for work) and family functioning (aOR = 1.72; *p* < 0.001; higher consumption in those with severe family dysfunction).

**Table 4:** Association between risky alcohol consumption and sociodemographic, occupational and family characteristics, stratified by sex

**Variables**		Unadjusted OR	95% CI	p	aOR^a^	95% CI	p
**MEN**							
** Age**	17–20	1.47	1.19–1.82	< 0.001	1.66	1.18–2.35	0.004
	21–24	1.48	1.16–1.88	0.002	1.67	1.15–2.43	0.007
	> 25	1			1		
** Type of residence**	University housing	1.10	0.94–1.29	0.243	1.00	0.75–1.34	0.972
	Rented apartment	0.60	0.53–0.67	< 0.001	0.58	0.48–0.70	< 0.001
	Family / own home	1			1		
** Occupation**	Studying and looking for work	1.26	0.97–1.65	0.085	1.31	1.04–1.66	0.020
	Studying and employed	1.27	1.02–1.38	0.032	1.64	1.21–2.22	0.001
	Studying	1			1		
** Family functioning**	Mild Dysfunction	1.29	1.04–1.59	0.019	1.31	1.05–1.62	0.014
	Severe Dysfunction	1.69	1.23–2.32	0.001	1.72	1.25–2.37	< 0.001
	Normal Functioning	1			1		
** Family history of alcohol**	Yes	1.25	1.03–1.52	0.026	1.19	0.97–1.46	0.091
**WOMEN**							
** Age**	17–20	1.80	1.34–2.42	< 0.001	1.72	1.26–2.37	0.001
	21–24	1.58	1.12–2.21	0.008	1.38	0.97–1.95	0.069
	> 25	1			1		
** Type of residence**	University housing	1.17	0.96–1.41	0.112	1.01	0.84–1.22	0.904
	Rented apartment	0.57	0.49–0.65	< 0.001	0.54	0.47–0.63	< 0.001
	Family / own home	1			1		
** Occupation**	Studying and looking for work	1.12	0.96–1.30	0.045	1.20	1.16–1.61	0.026
	Studying and employed	0.78	0.62–0.99	0.135	0.98	1.02–1.40	0.862
	Studying	1			1		
** Family functioning**	Mild Dysfunction	1.35	1.15–1.59	< 0.001	1.37	0.76–1.26	< 0.001
	Severe Dysfunction	1.69	1.38–2.07	< 0.001	1.74	1.41–2.14	< 0.001
	Normal Functioning	1			1		
** Family history of alcohol**	Yes	1.16	1.01–1.33	0.041	1.12	0.97–1.30	0.119

^a^Adjusted Odd Ratio for age, type of residence, occupation, functioning family and family history of alcohol. 95%CI = Confidence Interval 95%

Regarding the factors associated with BD, the logistic regression model reveals that BD consumption in men was significantly related to the place of residence (aOR = 0.64; *p* < 0.001; with lower consumption in those in a rented apartment), occupation (aOR = 1.37; *p* = 0.002; higher consumption in those studying and looking for work) (Table 5). The adjusted analysis also shows that BD consumption in women was related to age (aOR = 1.58; *p* = 0.001; with higher consumption in respondents aged 17–21 years), place of residence (aOR = 0.54; *p* < 0.001; with lower consumption in those in a rented apartment), and occupation (aOR = 1.21; *p* = 0.001; higher consumption in those studying and working).

**Table 5:** Association between binge drinking and sociodemographic, occupational and alcohol-related characteristics, stratified by sex

**Variables**		Unadjusted OR	95% CI	p	aOR^a^	95% CI	p
**MEN**							
** Age**	17–20	1.36	1.18–1.57	< 0.001	1.28	0.98–1.69	0.074
	21–24	1.47	1.24–1.74	< 0.001	1.29	0.95–1.75	0.101
	> 25	1			1		
** Type of residence**	University housing	0.95	0.83–1.08	0.411	0.82	0.63–1.05	0.117
	Rented apartment	0.58	0.53–0.63	< 0.001	0.64	0.54–0.748	< 0.001
	Family / own home	1			1		
** Occupation**	Studying and looking for work	1.33	1.10–1.60	0.003	1.37	1.12–1.66	0.002
	Studying and employed	1.10	0.87–1.38	0.440	1.28	0.99–1.67	0.061
	Studying	1			1		
** Family functioning**	Mild Dysfunction	1.12	0.93–1.34	0.221	1.13	0.94–1.35	0.199
	Severe Dysfunction	0.95	0.71–1.27	0.732	0.95	0.71–1.27	0.745
	Normal Functioning	1			1		
** Family history of alcohol**	Yes	1.02	0.867–1.22	0.772	0.99	0.83–1.17	0.898
**WOMEN**							
** Age**	17–20	1.57	1.28–1.94	< 0.001	1.58	1.30–1.92	0.001
	21–24	1.49	1.25–1.78	< 0.001	1.51	1.21–1.87	0.069
	> 25	1			1		
** Type of residence**	University housing	1.00	0.86–1.16	0.992	0.91	0.79–1.06	0.904
	Rented apartment	0.55	0.50–0.61	< 0.001	0.54	0.49–0.60	< 0.001
	Family / own home				1		
** Occupation**	Studying and looking for work	1.06	0.91–1.24	0.436	1.21	1.07–1.36	0.001
	Studying and employed	1.09	0.98–1.22	0.107	1.36	1.15–1.61	0.000
	Studying	1			1		
** Family functioning**	Mild Dysfunction	0.99	0.76–1.05	0.946	0.99	0.88–1.12	0.934
	Severe Dysfunction	0.89	0.76–1.05	0.165	0.91	0.77–1.07	0.270
	Normal Functioning	1			1		
** Family history of alcohol**	Yes	0.97	0.87–1.07	0.509	0.99	0.88–1.12	0.538

^a^Adjusted Odd Ratio for age, type of residence, occupation, functioning family and family history of alcohol. 95%CI = Confidence Interval 95%

## Discussion

The results of this study show that the pattern of risky alcohol consumption in university students is significantly associated with severe dysfunctional family structures, although no significant association is observed between risky alcohol consumption, binge drinking pattern and the presence of a family history of alcohol consumption mentioned by these students, when it is adjusted by sociodemographic and occupational factors.

Differences were observed when analyzing risky alcohol consumption stratified by sex: male students between 17 and 24 years of age who studied and worked or were looking for a job and who had a moderate or severe dysfunctional family structure had higher risky alcohol consumption. Female students between 17 and 20 years old who studied and looked for work and who had a moderate or severe dysfunctional family structure presented a higher risky alcohol consumption. Greater use of the binge drinking pattern was identified in male students who were also looking for work and in female students between 17 and 20 years old, who studied and worked or were in search of employment. Although there is no information available related to the pattern of alcohol consumption in private universities, our results could be extrapolable to Spanish private universities since the sociodemographic and occupational factors defined in our sample are similar to the results obtained in Spanish private universities (higher percentage of students between 21–24 years old, female, enrolled in careers in Social and Legal Sciences, followed by students who are pursuing careers in Health Sciences) [22].

There are multiple factors that influence college students’ alcohol consumption: demographic factors, personality type, personal drinking history, expectations when drinking, reasons for drinking alcohol, type of activity, academic participation, and family and social influence [23, 24]. In this article we focus primarily on the study of family influence and social support, personal history of alcohol, demographic factors and the type of activity performed by students.

In general, an environment in which alcohol consumption is encouraged and perceived as positive and normal tends to have more drinkers than peer groups where excessive alcohol consumption is not encouraged [7]. The relationship between family and alcohol consumption is not limited to the already established causality; there is another aspect, no less important that refers to the importance of this pathology in family interactions, and to the dysfunctional relationship dynamics that are created due to this problem [7]. Unstable and incoherent family and living environment factors (for example, transitional living conditions, inconsistent care, violence) resulting from substance use that caregivers have linked to the incidence of psychological and emotional development problems among their children [7].

There is a correlation between family functioning and the presence of addiction, such as alcohol consumption, showing the need for family support [25]. Similarly, Sánchez Queija et al., [26] point out that affective family relationships play a significant role in the prevention of substance use, like alcohol or tobacco, in adolescence and young adults. Thus, individuals who have received care and support during childhood, and enjoy a more cohesive family environment during adolescence and adulthood, showed less substance use. Individuals who start using in those years do not reach the level of substance use observed among those who have grown up in less favorable family contexts.

Severe family dysfunction is related to an increase in standard drinking units of alcohol / week, an increase in smoking, and in the use of illegal drugs [27]. Like the literature, our results show that there is a significant association between students' risky alcohol consumption pattern and dysfunctional family structure, with no such significance observed in the binge drinking pattern.

There is mixed scientific evidence on the association of family history of alcohol consumption with alcohol consumption, among which is intensive consumption [28–30]. and alcohol dependence [31]. A meta-analysis carried out in university students reveals that family history appears to have significant small to medium effects on the consequences of alcohol, symptoms of alcohol use disorder, and the participation of other drugs in samples of higher education students [32]. In contrast, small effects (many not significant) were found for consumption alone.

This suggests that college students with a family history may not drink more overall, but those who use alcohol or drugs may be more susceptible to problematic use.

The current study has some limitations: the questionnaire used has not yet been validated. However, this questionnaire consists of validated questions and scales from previously validated national questionnaires, like the Spanish National Health Survey [16] or the EDADES [3] survey, among others. The participation rate is around 3–5% depending on the university, taking into account that the project is a dynamic cohort that involves not only a baseline survey but also a follow-up over time and that participation is entirely voluntary, with no financial or other compensation for the collaboration. Another limitation of our study is its design. Although cross-sectional studies can determine prevalence, they cannot establish causality between alcohol consumption patterns and the other variables considered, especially family support. However, based on biological plausibility and the results of previous longitudinal studies, the observed trend, at least in the case of family support, could be correct.

## Conclusion

Risky alcohol consumption in university students is associated with family dysfunction, unlike the binge drinking pattern, where there is no such association. The findings of the study show the importance of creating prevention programs focused on the family approach in university students, which include alcohol screening in the population with a family history of this substance, as well as greater social support from the health services.

## Supplementary Information

**Additional file 1: Suplemmentary Table.** UniHcos Project raw data.

## Data Availability

The dataset is available on supplementary material.

## Abbreviations

AUDIT: Alcohol Dependent Disorders Identification questionnaire
BD: Binge Drink
CI: Confident Interval
EDADES: Survey on Alcohol and Drugs in Spain
ENS: National Health Survey
OR: Odds Ratio
SD: Standard Deviation
WHO: World Health Organization
